# Unveiling the heterogeneous utilisation of the same digital patient management platform: case studies in primary healthcare in Sweden

**DOI:** 10.1186/s12913-024-11287-3

**Published:** 2024-07-22

**Authors:** Susanne Frennert, Christofer Rydenfält, Mirella Muhic, Gudbjörg Erlingsdóttir

**Affiliations:** 1 Department of Design Sciences, Lund University, Lund, Sweden; 2 Department of Informatics, Umeå University, Umeå, Sweden

**Keywords:** Digital Patient Management, Primary Healthcare, Implementation, Case Studies, Healthcare Professionals’ Perspectives

## Abstract

**Background:**

The utilisation of digital technology in primary healthcare, particularly digital patient management platforms, has gained prominence, notably due to the global pandemic. These platforms are positioned as substitutes for face-to-face consultations and telephone triage. They are seen as a potential solution to the escalating costs associated with an aging population, increasing chronic conditions, and a shrinking healthcare workforce. However, a significant knowledge gap exists concerning the practical aspects of their implementation and their effect on the utilisation of digital patient management in primary healthcare.

**Methods:**

This study addresses this gap by conducting a comprehensive analysis of three case studies involving the implementation of a specific digital patient management platform. Over a period of three years, we examine how the practicalities of implementation shape the adoption and utilisation of a digital patient management platform in three different clinics.

**Results:**

Our findings revealed that differences in implementation strategies directly influenced variations in utilisation. The successful utilisation of the platform was achieved through a bottom-up decision-making process that involved the employees of the primary healthcare clinics. Onsite training, close collaboration with the eHealth provider, and a structured patient onboarding process played crucial roles in this utilisation. In contrast, a top-down approach at two of the primary healthcare clinics led to limited utilisation of the platform into daily workflows. Furthermore, making the platform a part of everyday work meant putting accessibility, by working as a team of physicians, at the forefront of continuity of care, with patients being managed by their designated physician. Additionally, it was observed that digital patient management proved most effective for addressing simple patient issues such as skin rashes, rather than complex cases, and did not reduce the demand for phone triage.

**Conclusion:**

Only one of the three clinics studied effectively integrated digital patient management into its daily operations, and did so by aligning objectives among management and all categories of healthcare professionals, employing a bottom-up decision-making process, collaborating with the eHealth service provider for regular platform adjustments to clinic needs, and implementing active patient onboarding. This sociotechnical integration resulted in high platform utilisation. In contrast, the other two clinics faced challenges due to incoherent objectives among diverse healthcare professional employees and top management, a top-down decision-making approach during implementation, limited collaboration with the eHealth service provider, and passive patient onboarding. The findings indicate that these factors negatively affected utilisation and led to low platform adoption as well as disrupted the sociotechnical balance.

**Supplementary Information:**

The online version contains supplementary material available at 10.1186/s12913-024-11287-3.

## Background

The potential to use digital patient management, such as digital triaging and video consultations, as a substitute for in-person primary healthcare consultations and phone triaging has been widely acknowledged [1]. The digital transformation of primary healthcare is portrayed as a solution to address the rising costs associated with an aging population, increasing chronic conditions, and reduced workforce. It represents a dominant sociotechnical imaginary [2], and many countries have promoted and invested in the digital care imaginary (e.g., United Kingdom, Australia, Denmark and more) [3–6]. One of these countries is Sweden, which since 2006 has had three national eHealth strategies [7–9]. All these strategies express similar values and arguments: innovation, patient-centred care, patient engagement, efficiency, availability, accessibility, equality and privacy [10]. The most recent eHealth strategy, in 2016, states that Sweden by 2025 ‘will be best in the world at using the opportunities offered by digitisation and eHealth, making it easier for people to achieve good and equal health and welfare, and to develop and strengthen their own resources for increased independence’ [9].

Amidst this digital transformative landscape, our study aimed to investigate a fundamental question that has received limited attention: *why does the practical application of digital patient management result in varying patterns of usage across different primary healthcare practices?* This question is of utmost importance, as the outcomes of these implementations can deeply affect patient care, healthcare professionals’ working environment, and the healthcare system as a whole.

Despite the prominence of eHealth strategies, and the acceleration of digital technologies in healthcare due to the recent global pandemic [11, 12], a critical gap persists in our understanding of technology implementation in primary healthcare. A scoping review revealed that there is limited evidence on the actual effects of digital patient management in primary care on the working environment [13, 14]. What we do know is that phone triaging, predominantly performed by nurses, has often been the source of considerable stress [15]. In contrast, studies on digital patient management have generated contradictory results: digital patient management has been credited for providing nurses with greater flexibility and autonomy [16], while it has been criticised for increasing nurses’ workload [17], and loss of job control [18].

This apparent contradiction may be explained by the fact that digital patient management platforms are not isolated entities but part of broader sociotechnical systems, including not only technology, but also people, organisations, norms and values [19]. According to sociotechnical systems theory, the same technology can yield different outcomes because technology and technical aspects interrelate with social and organisational aspects. As such, working with digital patient management is a social process in which healthcare professionals need to match the problem (sick individuals) to a non-standardised solution, as dealing with sick individuals requires ad hoc and pragmatic responses, which often entails collaboration with other healthcare professionals [20]. When new technology is introduced, healthcare professionals as well as patients need to make sense of the technology and negotiate regarding the ways in which it fits their needs [21]. Theories on technology-in-practice, show that healthcare technologies are not only shaped by healthcare professionals but also mediate care praxis [22]. Thus, a growing body of literature on digital patient management highlights the importance of extending the scope beyond the impact of digital patient management per se, to understand what factors facilitate its implementation [23–26] and healthcare professionals’ lived experiences of its impact on their work and working environment [27].

Thus, the purpose of this paper is to offer an in-depth exploration of the implementation of a common digital patient management platform at three primary healthcare clinics – a study extending over a three-year period. In one clinic, digital patient management became seamlessly integrated into daily care practices, while in the other two, it remained underutilised. The heart of our investigation seeks to unravel the reasons behind these disparate patterns of utilisation. By doing so, we hope to provide valuable insights for healthcare practitioners, guiding them to circumvent potential pitfalls and facilitate the successful adoption of digital patient management in the realm of primary care.

## Method

This qualitative study applied a multiple case study design with three different cases [28]. Below we will first present the digital patient management platform being implemented and give a brief overview of the case organisations. We will then describe the data collection and analysis process.

### The digital patient management platform

The digital patient management platform includes automatic triage, e-consultations (via chat and video), and case management [29–31]. The patients access the platform online via a computer, tablet or smartphone, describe their symptoms and receive automated questions depending on their input; the system finalises a medical report that can be viewed by the healthcare professionals at the primary healthcare clinic. Initially the medical report is reviewed by a nurse, who either handles the patient request or forwards it to a physician, psychologist or physiotherapist. Healthcare professionals have an internal chat function in which they can discuss patient cases and actions needed. Communication with patients, as well as appointments, can occur synchronously or asynchronously in the form of digital or physical meetings with different categories of healthcare professionals.

### The case organisations

The cases are three primary healthcare clinics located in southern Sweden. Two are owned by a private healthcare provider and (see Table 1, clinics B & C) turned out to be very similar, especially in regard to the objectives of implementing the platform, communication about the platform with patients, training, responsibility of the change team, leadership engagement, and how the nurses’ and physicians’ work was organised, while the other clinic belongs to a Christian foundation (see Table 1, clinic A). This primary healthcare clinic differed from the other two, especially in terms of coherence with its overall communication with patients about the digital patient management platform, responsibility of the change team, training, leadership engagement, and how the nurses’ and physicians’ work was organised.

**Table 1:** The cases

	Primary Healthcare Clinic A	Primary Healthcare Clinic B	Primary Healthcare Clinic C
Number of listed patients	9000	7500	6500
Number of employees	35	15	15
Interviews, round 1	4 nurses 3 physicians 1 rehabilitation coordinator 1 psychologist 1 head of management 1 line manager	5 nurses 1 physician 1 head of management 1 medical secretary	4 nurses 3 physicians 1 head of management 1 medical secretary
Interviews, round 2	3 nurses 4 physicians 1 rehabilitation coordinator 2 psychologists 1 medical secretary 1 head of management 1 line manager	6 nurses 2 physicians 1 head of management 1 medical secretary	3 nurses 1 head of management
Interviews, round 3	5 nurses 1 head of management	3 nurses 4 physicians 1 head of management	3 nurses 2 physicians 1 head of management
**Interviews in total**	30	26	19
Observations	3 full day	11 half days	6 full days

### Data collection

The multiple-case study was carried out between 2020 and 2023 using a series of in-depth semistructured interviews and observations (see Table 1 and Appendix A). The selection of participants was facilitated by the head of management at each clinic. The inclusion criteria were healthcare professionals who worked with patients and would use the digital patient management platform when implemented. The researchers attended an introductory meeting with the head of management and the clinic’s employees, during which the project and methodology were presented. All employees were asked about their willingness to participate and to inform their managers of their decision. The managers then provided the researchers with contact details of those who wanted to be interviewed and the nurses who agreed to be observed. Subsequently, the participants received written information about the project and signed informed consent forms. As the project spanned over three years, many of the initial participants left the clinics and we had to contact the head of management throughout the years for contact details of potential new participants, whom we then contacted via email. Those who agreed to participate were interviewed.

Over the course of the three-year project, we conducted interviews with psychologists, physicians, rehabilitation coordinators, heads of management, line managers, and medical secretaries before, during, and after the deployment of the digital patient management platform. The interviews lasted between 15 min and one hour, depending on the healthcare professionals’ experience with the platform. In total, 75 interviews were conducted. All interviews were recorded and transcribed.

#### Timeline

In Spring 2020, Clinic A implemented the digital patient management platform. The first round of interviews was conducted at the beginning of 2020 via videoconferencing. Due to pandemic restrictions, observations could not be conducted prior to the platform’s implementation. The second round of interviews at Clinic A was conducted in Spring 2021 through videoconferencing and the third round of interviews took place in 2022. In Spring 2022, observations were conducted involving three nurses who worked half a day with phone triage and half a day with patient errands on the digital platform.

Clinics B and C implemented the digital patient management platform in Autumn 2021. The first round of interviews at clinics B and C was carried out in Spring 2021 via videoconferencing. Observations were performed in September 2021, before the platform’s implementation, coinciding with the lifting of pandemic-related restrictions in Sweden [32]. Three nurses were observed at both clinic B and clinic C. At clinic B, each nurse was observed for half days, whereas at clinic C, observations lasted full days. The reason for half days at clinic B was due to the management’s decision on how long the observations were allowed to last. The second and third rounds of interviews at clinics B and C were conducted in Spring 2022 and Spring 2023. A second round of observations was conducted in 2023 at both clinics. The objective was to observe the same three nurses who were initially observed prior to the platform’s implementation while engaged in phone triage, but in this round of observations, they were using the digital patient management platform. At clinic B, all nurses from the initial observations had departed and we had to recruit new nurses. These new nurses were observed first while performing phone triage (over four half days) and subsequently while using the digital platform (over another four half days). At Clinic C, the same three nurses from the initial round of observations were observed again.

During the phone triage observations, focus was on nurses’ verbal communication with patients, the frequency and nature of interruptions, interactions with colleagues, time for handling patient errands, and observable emotional responses such as frustration and satisfaction. The digital triage observations focused on the same factors as with the phone triage observations but with an additional emphasis on written communication with patients and the usage of the digital platform. The observations resulted in fieldnotes.

#### Analysis

We employed hermeneutic interpretive phenomenology to make sense of how healthcare professionals experienced the implementation of the digital patient platform in different clinics [33]. The primary focus of our analysis was to gain insights into the practical aspects of the implementation from the healthcare professionals’ point of view. The substantial volume of data was sequentially analysed throughout the three years (see Table 1). We wrote summaries of central concerns, which were redefined based on the data collected during subsequent interviews and observations. We reviewed and refined these summaries throughout the study, identifying key aspects, such as how patients learned about the digital patient management platform at the healthcare clinic, implementation strategies, such as forming a team of super users, the type of training received by employees, engagement from management, and how nurses and physicians planned to incorporate digital patient management. These aspects formed the foundation for case descriptions. We selected quotations to illustrate the lived experiences of healthcare professionals. The core findings concerning the practicalities of implementation and their variations were shared with stakeholders at national conferences, and further refined through discussions with stakeholders.

### Description of the cases

The implementation process at the three primary clinics in our study varied. Table 2 provides descriptions of the clinics, illustrating the similarities and differences in terms of objectives for implementing the digital patient management platform, patient communication about the platform, change team, training, leadership engagement, nurses’ work and physicians’ work.

**Table 2:** Overview of cases

	Primary Healthcare Clinic A	Primary Healthcare Clinic B	Primary Healthcare Clinic C
**Objectives for implementing the platform**	To reduce stress among nurses, address competition from telemedicine providers, and enhance patient accessibility.	To increase patient accessibility and reduce stress, particularly for nurses.	To increase patient accessibility and reduce stress, particularly for nurses.
**Patient communication about the platform**	Patients received a link to the platform when phoning the clinic. Nurses and physicians initiated contact with patients through the platform.	Patients received a link to the platform when phoning the clinic. Nurses and physicians only handled patient requests initiated by the patient through the platform.	A link to the platform was available at the clinic’s website. Nurses only handled requests initiated by patients through the platform. Physicians seldom communicated with patients through the platform.
**Change team – team of super users**	One physician and two experienced nurses.	Head manager and a medical secretary.	Head manager, a nurse, and a physician – although main responsibility delegated to nurse.
**Training**	Initial onsite training sessions for all staff, given by the eHealth service provider. Following deployment, new employees acquired platform skills through hands-on experience and observations of colleagues.	Initial online training sessions for all staff, given by the eHealth service provider, and one person responsible for implementation, hired by the private healthcare provider. Following deployment, new employees acquired platform skills through hands-on experience and observations of colleagues	Initial online training sessions for all staff given by the eHealth service provider and one person responsible for implementation (as in clinic B) hired by the private healthcare provider. Following deployment, new employees acquired platform skills through a video.
**Leadership engagement**	Decision to employ and utilise the platform was a joint decision by the head manager and the staff.	Top-down decision by private healthcare provider to deploy the platform. Head manager actively involved; however, left the clinic after one year.	Top-down decision by private healthcare provider to deploy the platform. Head manager delegated deployment responsibility to one nurse.
**Nurses’ work**	Initially, one nurse worked in the platform in the morning and one in the afternoon. However, this changed over time, as the nurses wanted to work full days in the platform. Depending on the amount of patient requests in the platform, one or two nurses worked full days in it.	One nurse was responsible for handling patient requests in the platform from 8–9 am, after which the same nurse worked in the platform as time allowed, parallel to giving telephone advice.	Three nurses worked in the platform in parallel with giving telephone advice. They handled most patient requests themselves, or booked a physical appointment with the physicians.
**Physicians work**	Initially, all physicians, except one who did not want to use the platform, had morning and afternoon slots for attending patient requests in the platform. During follow-up interviews, patient requests were mainly addressed by one designated physician. Physicians took turns assuming responsibility and had daily allocated time for patient requests. One physician was always available through the platform.	Nurses put 15 min into physicians’ schedule after receiving a patient request via the platform. Requests were handled by the patient’s family physician.	Nurses allocated 15 min in physicians’ schedules after receiving patient requests through the platform. Requests were managed by the patient’s designated physician. However, physicians rarely received patient requests.

#### Primary healthcare clinic A

Clinic A, a middle-sized clinic with approximately 9000 listed patients and 35 employees, began exploring ways to utilise digital technology for their patient interactions in 2019. They identified phone triaging as a source of stress, particularly for nurses, as they received many calls and had a backlog of patients. In response, one of the physicians suggested piloting a digital patient management platform. After conducting exploration and consulting with an eHealth service provider, the healthcare professionals and the clinic manager jointly decided to pilot the digital patient management platform for several months to evaluate its effectiveness and user experience.

Prior to the pilot, the manager received implementation materials from the eHealth service provider, based on Kotter’s approach to change management [34]. The eHealth service provider stressed the need to establish *a clear vision* for the digital patient management platform’s implementation, focusing on optimising patient flow, relieving nurses of phone triage duties, and enhancing knowledge exchange among healthcare professionals. They also recommended forming a team of ‛super users’, comprising different professions, to streamline workflows. Aligning the clinic’s schedule with the platform and providing resources for staff adaptation were key priorities. Effective communication channels for issue resolution and patient promotion of the platform were emphasised, with a focus on the correlation between increased platform usage and perceived benefits.

Primary healthcare clinic A embraced these recommendations, acknowledging the importance of reducing nurse workloads and improving patient accessibility to compete with online doctor services and meet patient expectations. A team of super users, comprising a physician and two experienced nurses, was established to oversee implementation. The physician assumed an informal leadership role in implementing and advocating for the digital patient management platform. Physical training sessions were arranged for all employees, allowing them to express their hopes and concerns before implementing the digital platform. Many employees expressed that the platform was easy to learn and use; one employee noted:

I sat with colleagues and learned. It is a pretty simple system [the digital patient management platform]. There are not many buttons to press, so you actually learn the system very quickly.

Employees actively participated in reshaping their workflow, advocating for the platform to patients and colleagues, emphasising benefits such as increased patient accessibility and reduced workload, particularly for nurses. As one nurse at primary healthcare clinic A explained:

There are many people I refer to our chat [the digital patient management platform] if I have the opportunity because it makes it easier for me, and I can make an assessment faster … a lot of skin rash assessments and things where the patient can send in pictures so you can see, instead of just hearing their explanations.

Regular meetings were held before, during, and after implementation, providing a forum for employees to express their expectations, experiences, and concerns. The physician on the team of super users presented data on patient flow and patient experiences during these meetings, helping the team identify obstacles. The eHealth service provider regularly adjusted the digital patient management platform based on feedback from clinic A.

Initially, nurses worked for half a day on the digital platform. However, it became evident that they needed to work full days due to the drawn-out nature of asynchronous communication. Working full days allowed them to complete most patient requests, as asynchronous communication required them to manage multiple patient requests concurrently over longer time periods. To further enhance the workflow, patient requests to physicians were handled by a team of physicians instead of being assigned to the patient’s designated physician. The team of physicians was allocated specific time slots to work via the platform, ensuring that patient requests were always attended to, even if an individual physician was unavailable due to physical appointments, illness, or other reasons. This team approach minimised the risk of missed patient requests, and ensured smooth handling of requests. The head management at clinic A explained:

*We are divided into teams*,* and there is always someone from the team who is in the chat [the digital patient management platform] every day and can make prescription requests and patient requests. Initially*,* we saw that there was a concern about handing over a patient errand to a designated physician who may be absent for a day*,* and then the physician becomes sick for a week … patient requests were left hanging … now we work very team-based … you transfer the patient requests to the team … you never transfer them [patient requests] to a specific physician.*

Employees and management at clinic A emphasised the benefits of working with patient requests through the platform, including flexible working arrangements, reduced stress, and improved work-life balance compared to phone triaging. Asynchronous communication allowed for more flexibility, and some nurses found it a welcome relief compared to the demanding nature of phone triaging, where immediate responses were needed. One nurse at clinic A explained:

*It [the digital patient management platform] is still more flexible. If you work with patient requests through the platform for a day*,* you are more flexible. You can take a break and do something else … you do not have to stick to exact times. That is why it has been quite easy to work from home … it can be difficult to sit and make phone calls at home with sick children*,* but working with the platform has worked quite well*,* better in any case*,* just because you can still text someone on the side.*

Similarly, another nurse colleague highlighted the differences between phone triage and the platform:

I think that the phone is more demanding … you need to be slightly more engaged. The patient notices if you are not really listening to the questions you’re getting and so on. In the chat, you can express yourself, however you want. For better or worse, you don’t convey emotions in the same way in the chat … in the chat, you can read a question and then think for a while before giving an answer. You can consult colleagues more easily in a different way. On the phone, you get a question. And then you need to listen and respond immediately … it requires slightly more from you, so to speak.

However, some nurses at clinic A found working with patient requests through the platform to be stressful because there was no limit to the number of requests that could pile up, unlike phone triage, where the number of requests was limited to the time slots of that day. On the other hand, physicians at clinic A found that working through the platform reduced interruptions, as nurses could communicate with them through the platform, rather than interrupting their work with in-person visits.

At the end of the three-year study, the digital patient management platform became an integral part of the daily work of nurses, psychologists, and physicians (see Table 3). They scheduled dedicated time to work on the platform and felt that it supported their independence, and better planning of their work. The platform was experienced as less stressful and provided opportunities for recovery and flexible working arrangements. Healthcare professionals actively initiated contact with patients by using the platform for communication, sharing forms, test results, and follow-up appointments.

However, the clinic’s vision of reducing nurses’ workloads and increasing their availability to patients faces challenges. The significant increase in patient inquiries and demands during the pandemic and postpandemic periods added to employee workloads and pressure. Asynchronous communication on the digital platform, involving ongoing patient requests that could span hours or even days, presented unique challenges for nurses who had to manage multiple patient requests concurrently. This contrasted with phone triage, where patient inquiries and requests were typically resolved within minutes. Additionally, the clinic experienced high employee turnover, further straining the remaining staff. Nevertheless, healthcare professionals and management actively engaged in institutional work to advocate for the platform’s benefits, reshaping primary care work at the clinic.

**Table 3:** Average number of patient requests in the platform for one week

	Primary Healthcare Clinic A	Primary Healthcare Clinic B	Primary Healthcare Clinic C
Introduction of the platform	Spring 2020	Autumn 2021	Autumn 2021
First round of interviews	Spring 2020	Autumn 2021	Autumn 2021
Average number of patient requests in the platform, during one week	210	33	10
Second round of interviews	Spring 2021	Spring 2022	Spring 2022
Average number of patient requests in the platform during one week	266	24	5
Third round of interviews	Spring 2022	Spring 2023	Spring 2023
Average number of patient requests in the platform during one week	217	17	14

#### Primary healthcare clinic b

Clinic B, a small-sized clinic with approximately 7500 listed patients and 15 employees, implemented the digital patient management platform in 2021. The decision was made by a private healthcare provider overseeing multiple clinics in the southern region of Sweden, without involvement from local managers. The main objectives the private healthcare provider was to reduce employee workload and enhance patient accessibility. However, the nurses at clinic B were initially skeptical about the necessity of the platform. They raised concerns that increased availability might prioritise younger patients with less serious conditions over older adults with more pressing health needs.

Despite their reservations, some acknowledged the potential benefits of digitalisation in terms of improving patient accessibility. However, in their daily practice, they did not perceive a need for change and thus did not actively advocate for the platform to patients or colleagues. They considered working with the platform an additional burden but believed it could benefit patients by providing an additional way to contact primary healthcare. One physician at clinic B stated:

High availability for patients, of course. It is fast and smooth for them. The drawbacks are that as physicians, we don’t have time … it becomes too much for us because we have to log in to three, four, five different places. In addition to 1177 [the national healthcare platform], the digital patient management platform and the electronic healthcare records, there are also other things we have to do … it just becomes a lot of work … that is time-consuming. I really wish this [all digital systems] could be narrowed down somehow. But for patients, it’s great.

To implement the platform, the private healthcare provider offered online training sessions. The large-scale implementation of the platform involved providing online training sessions to all primary healthcare clinic employees in southern Sweden, which were conducted by the eHealth service provider and a centrally appointed implementation manager hired by the private healthcare provider. During these training sessions, the eHealth service provider emphasised the importance of change management, using Kotter’s approach, as seen in clinic A. Clinic B’s healthcare professionals received this online training and followed Kotter’s change management model, which included the appointment of a team of super users, although the team’s effectiveness was limited due to busy schedules, staff turnover, the pandemic, and other challenges.

Initially, one nurse at clinic B started handling patient requests in the morning, switching between the digital patient management platform and phone triaging. However, nurses found this approach inefficient due to the lack of real-time feedback on the platform, which led to interruptions from phone triaging. This required them to repeatedly revisit earlier communications with patients on the platform, making the process disjointed and time-consuming. When patients responded promptly and the requests were straightforward, the platform’s efficiency was comparable to that of phone triage. Physicians at clinic B experienced similar challenges, with patient requests routed to their designated physician and a 15-minute time slot allocated for handling platform requests. However, the physicians noted the inconsistency of patient requests and the difficulty of accommodating patients within their busy schedules. This resulted in limited time for each task, particularly when requests required clarification or when patients did not respond immediately. One physician explained:

Honestly, I don’t like the digital patient platform. And it is because, firstly, there is no designated time for it. So, no matter how many cases there are, I do not have time for each one. That is how it is. Secondly, some cases are for prescription renewal, and then it is manageable, but sometimes the patient wants to talk to a physician, and I have not designated time for that, so it becomes very difficult for me to find time in the schedule when it is already full [the schedule]. And the problem is that it becomes tedious because it takes time to write to the patient, get a response, and then it is also time-consuming because patient requests that initially seem straightforward become more and more complex as patients bring up more and more details, and it becomes a bit harder to limit the patient when it is in text form or chat. In the end, a physical visit may have been preferred as I may solve the problem in 15 min during a physical visit.

Another physician highlighted:

I send letters, make phone calls. Occasionally, I sent a text message via the electronic healthcare records but not through the actual digital patient management platform. Because if I have to go into the digital patient management platform, it’s extra steps. Then I have to go into the link, and open it … We have the electronic healthcare record, Pascal [the medication record], and NPÖ [national patient overview] … we have a lot of other things, and then I feel like … I don’t want to go into the digital patient management platform and respond … It’s an extra step to go into another system and then document … if I can call the patient and write notes in the electronic healthcare record at the same time, I might finish in half the time.

As the quotes above illustrate, employees at clinic B approached the platform reluctantly, viewing it as an additional system to manage. During busy hours, they felt it added to their workload. While they found the platform manageable for ‛easy’ tasks, such as prescription renewals, they did not consider it their preferred solution. They believed that the platform did not enhance the quality of their work environment. As one nurse at clinic B expressed:

*It doesn’t help me in my profession*,* and it doesn’t help me provide better care for the patients.*

The internal chat features introduced during training were not widely used, with colleagues opting for oral communication to resolve issues. Video-conferencing with patients was not perceived as beneficial and was seldom utilised. Most nurses resorted to a copy-and-paste method for record-keeping in the electronic healthcare records due to time constraints. Some nurses were uncertain about the preferred method, leading them to both copy and paste information and write summary assessments. One nurse at clinic B explained:

First you have to copy the text they [the patients] have written [in the digital patient management platform], and there’s a lot of clicking and pasting and clicking and pasting [into the electronic healthcare record], and then you have to write your own assessment too … it is cumbersome.

A physician at clinic B noted:

They [the nurses] paste everything in … when it’s just pasted in like that, it’s difficult to read and understand what it is really about and what the problem or question is.

By the end of the study, only a few patients were using the platform (see Table 3). Employees felt that patient adoption seemed to depend on phone availability, with more platform use occurring when phone availability was limited. Employees did not initiate patient contact through the platform but rather responded to patient requests that came in via the platform. They explained that management directives were to meet patients through their chosen communication channel. If a patient called, the clinic handled the request over the phone; if a patient used the platform, the request was addressed on the platform.

#### Primary healthcare clinic C

Clinic C, a small primary healthcare clinic serving approximately 6500 registered patients and staffed by 15 employees, followed a similar path to clinic B in implementing the platform. The decision to deploy the platform at clinic C was made by the same private healthcare provider that oversees clinic B, and it did not involve direct input from local management or employees. The primary reason behind this decision was to reduce workloads and improve patient accessibility. However, the nurses at clinic C did not agree and noted that they already had a high level of availability and that patients had not expressed a desire for digital care or increased accessibility.

In clinic C, healthcare professionals engaged in a series of online training sessions and adhered to Kotter’s change management model, which entailed the appointment of a team of super users as seen in clinic B. This group comprised the head manager, a nurse, and a physician. However, due to their demanding schedules, which were further heightened by factors such as staff turnover and the challenges stemming from the pandemic, the team of super users did not take a proactive role in driving the implementation process. The manager was preoccupied with overseeing the clinic, leading to the delegation of power of the implementation of the digital patient management platform to one of the nurses. Despite mandatory online training, employees lacked interest in the platform and participated in the online training sessions. As a result, the clinic initially decided to restrict platform usage to only nurses conducting phone triaging. The lack of engagement was narrated by all the healthcare professionals at the clinic, including the nurses. As one of the nurses explained,

*It* [the implementation of the digital patient management platform] *wasn’t really our decision* [the clinic]; *it came from higher up. We were controlled from above*,* so to speak. They wanted us to implement it*.

The nurses worked with the platform in parallel to working on the phone. During the study, the physicians became slightly more involved and used the platform, and hence only had a few requests during the three-year study period. The platform was not discussed in great detail at clinic C. During the introduction, the employees felt that it caused increased stress. However, there was a consensus among the healthcare professionals that the digital patient management platform itself was not the primary source of dissatisfaction. Instead, they recognised that the negative impact on their working environment was largely influenced by organisational factors, such as the heightened stress caused by increased pressure from phone triaging. This meant that nurses had to juggle both answering phone calls and attending to requests through the platform, while physicians had to balance their regular tasks and the additional demands from the platform.

The adoption of the digital patient management platform was somewhat impeded at clinic C by their interpretations of the Swedish national healthcare guarantee, which states that everyone who calls should be able to reach their healthcare clinic for advice and/or booking an appointment on the same day [35]. This is evaluated based on the proportion of patients who can make contact with their healthcare clinic on the same day during a measurement period. Clinic C interpreted the guarantee to be based solely on telephone contact, leading them to prioritise high accessibility via phone over rapid responses to digital contacts through the digital patient management platform. By the end of the study, clinic C had few patients who used the digital patient management platform (see Table 3). According to the employees, the platform did not significantly impact their work or working environment.

A quote from the head manager, who was also initially part of the team of super users, nicely summarises the platform’s implementation at clinic C by the end of the study. The head manager explained:

*Well*,* I must say that the implementation has been smooth and trouble-free*,* largely due to our excellent telephone accessibility. Telephone availability here is consistently at 98% and reaching us has always been easy. Consequently*,* there has not been much need for people to contact us through the digital patient management platform. Personally*,* I haven’t had to be involved in the implementation because it hasn’t been necessary.*

### Findings

As seen in the three cases presented, the practicalities surrounding the implementation of the same digital patient management platform in three primary healthcare clinics present different opportunities and challenges. By reflecting on these practicalities, we can gain insights into aspects that contribute to the successful integration and utilisation of such platforms. These practicalities concern a number of dimensions in which the cases differ, i.e., coherence, decision-making approaches, collaboration with eHealth service providers, training, patient onboarding, organisation of work, and distribution of patients. An overview of these dimensions can be found in Table 4. We will discuss them in the following sections.

**Table 4:** Differences and similarities between the cases which the same digital patient management platform was used

Dimensions	Clinic A	Clinic B	Clinic C
Objectives	Coherent between all stakeholders	Non-coherent	Non-coherent
Decision making	Bottom-up	Top-down	Top-down
Collaboration with provider	Close collaboration	No collaboration	No collaboration
Training	Onsite training	Mass online training	Mass online training
Patient onboarding	Active	Passive	Passive
Organisation: workflow	Standardised routines	Ad-hoc	Ad-hoc
Organisation: distribution of patients	Team of physicians	Designated physician	Designated physician

### Coherent versus non-coherent objectives

While all three primary healthcare clinics had similar objectives for implementing the patient management platform, only clinic A’s employees and management had a shared understanding of its necessity. In contrast, employees at clinics B and C questioned the objectives set by top management, as they did not find them meaningful and were unsure about how a new digital approach would benefit their work and professional identity. Van den Heuvel et al. [36] highlight the importance of *meaning making* and *change information* for employee’s adaptive behaviour in relation to organisational change. This indicates that to be able to act adaptively in relation to a change, one must also be able to *make meaning*, i.e., *make sense* of the change from a personal point of view, which did not occur in cases B and C. As pointed out by Rydenfält, Persson, Larsson, Johansson and Erlingsdóttir [37], a change that makes sense on one level might not do so on the local level where implementation occurs. This could be a problem related to framing, or it could be due to an actual lack of usefulness (or fit) of the change in relation to the organisation’s tasks. Framing is crucial for sensemaking, as sense is always made in relation to some kind of context or frame. As noted by Orlikowski and Gash [38], when the framing concerning a particular technology is incongruent, conflicts concerning implementation and use are likely to occur. In relation to the prevailing sociotechnical digital care imaginary, the results in cases B and C describe an incongruent framing [2].

### Top-down versus bottom-up decision-making approaches

A significant contrast between clinic A and clinics B and C was the origin of the decision to implement the platform. In clinic A, the initiative stemmed from the bottom-up approach, driven by nurses expressing their concerns about the stressful nature of phone triaging. The management, in collaboration with the employees, actively sought solutions to address the issue, leading them to pilot the digital patient management platform. After experiencing its benefits, they made the decision to integrate it permanently into their workflow. In contrast, in clinics B and C, the implementation of the platform was mandated by the central healthcare provider without the involvement of local primary healthcare clinics and employees. This top-down approach diminishes the participation and influence of employees in decision-making processes. According to past research, such top-down implementation approaches can lead to challenges and limited acceptance of new technologies within organisations [39–41], which was observed in clinics B and C.

Despite vastly different levels of success in implementing the digital platform, all clinics are illustrative examples of what, according to sociotechnical theory, is referred to as responsible autonomy [42, 43]. In practice, responsible autonomy refers to local leadership and adaptability at the group level, rather than work being organised from above by those not involved or related to the actual work situation and local context. The principle implies that it is better to have a simple organisation for complex tasks than the other way around. Here, a complex organisation would mean that the organisation has many levels and that decisions are made far away from the sharp end where the work is done. In clinic A, it is clear that responsible autonomy was a ruling principle as the change was locally driven. In clinics B and C, there was also signs of responsible autonomy. However, in their cases, it resulted in them (locally) choosing not to use the platform as they felt that it did not make sense given their framing of their work situation.

### Close collaboration with the eHealth services provider versus no collaboration

Furthermore, unlike in clinic A, where there was close collaboration with the eHealth service provider, clinics B and C lacked any contact or regular discussions with the eHealth service provider. This resulted in the absence of feedback loops and limited opportunities for employees to influence the functionalities and logics of the digital patient management platform. As a result, this led to a reduced sense of ownership among employees and a lack of perceived alignment between digital patient management and the specific needs and practices of primary healthcare clinics B and C. The findings indicate that active participation and meaningful engagement of employees play a paramount role in adoption of digital patient management. It emphasises the sociotechnical principle that technology implementation is not only about the technical aspects but also about how it aligns with the social context. This may be even more so in Nordic countries, since employees have an expectation to have their say in decisions that affect them [44].

### Onsite hands-on training versus mass online training

The training sessions in clinic A differed significantly from those in clinics B and C. In clinic A, the training was conducted onsite in the presence of the eHealth service provider, allowing for direct interaction and personalised guidance. On the other hand, in clinics B and C, the training sessions were conducted online, involving employees from multiple primary healthcare clinics owned by the same private healthcare provider. This approach, while potentially cost-effective, was shown to have negative implications for employees’ engagement and interest. Scmidt & Houst (2000) shed light on this phenomenon, demonstrating that small group discussions stimulate cognitive processes and positively influence individuals’ motivation to learn [45].

One can speculate that onsite small discussion groups could have been extremely valuable at clinics B and C, where the objectives set by top management were not agreed upon by the employees. During their mass online sessions, some employees were digitally present but not actively engaged. If they were compelled to actively learn how to use the platform through the social pressures of smaller groups, they might have had a chance to better understand the workings and benefits of digital patient management, making them more willing to utilise it. The situation at clinics B and C can be interpreted as a disconnect between the social and technical components of the system. The objectives set by top management did not align with the employees’ understanding, resulting in a lack of coherence in the sociotechnical system. The employees’ lack of engagement with the digital patient management platform during massive online sessions underscores the need for improved integration of social and technical elements. Using small onsite discussion groups may have addressed this imbalance. By creating smaller, more socially-oriented learning environments, they could have better integrated the social and technical components of the system.

### Active patient onboarding versus passive patient onboarding

Effective patient onboarding is contingent upon patient engagement and adoption of digital triaging. Without patient utilisation of the platform, its impact on the working environment remains limited. In clinic A, proactive measures were taken to steer patients towards using the platform, and healthcare professionals even initiated patient contact through the platform. However, such proactive patient engagement was not observed in clinics B and C. The volume of patients utilising the digital platform also plays a crucial role in the reorganisation of work routines to accommodate new digital practices, as seen in clinic A. The result indicates that the greater the patient volume is, the greater the potential for the integration of new digital routines within the primary healthcare clinic. From the lens of sociotechnical systems theory, this highlights the interplay between social factors (patient volume) and technical aspects (digital routines) and their impact on utilisation.

### Standardised routines versus ad-hoc approach

Another factor impacting the utilisation of the digital patient management platform was the ad-hoc approach taken by clinics B and C compared to the more standardised routines in clinic A. In clinics B and C, interviews revealed that healthcare professionals responded to patients using both phones and the platform, despite patients initiating digital contact. Additionally, nurses inconsistently copied the entire chat dialogue into the national healthcare records, while sometimes opting for a summary of their assessment. Some nurses regularly worked on the platform when time allowed, even though they were not scheduled to do so, while others did not. This lack of consensus on how to work with the platform resulted in different individuals interpreting and implementing their own working routines. In contrast, in clinic A, the management and healthcare professionals adhered to standardised routines.

### Team of physicians versus designated physician

One change made by clinic A to integrate digital patient management into its organisational context was the creation of a team of physicians and nurses dedicated to handling patient inquiries through the digital patient management platform, rather than leaving this responsibility to patients’ designated physicians. Due to the demanding schedules of physicians and the nature of asynchronous communication, this shift seems to be necessary. However, this raises important questions about the balance between continuity of care and accessibility. The results indicate that providing online access to care requires breaking the continuity of care with the patient’s designated physician. For simple tasks such as prescription renewal and assessing sores or rashes from photos, this was not a significant issue in terms of quality and efficiency of care. However, for more complex cases, the quality and efficiency of care may suffer without the continuity provided by the patient’s designated physician. One potential hypothesis drawn from these cases is that uncomplicated patient requests are well-suited for digital handling, while complex patient requests often require physical examination and continuity of care with the patient’s regular physician. Eriksson and colleagues conducted both an interview and focus group study and found that although digital triage platforms led to efficiency gains for patients with simple cases, the low uptake of the technology meant that it did not replace existing functions and routines, but rather added to them, resulting in a negative impact on overall efficiency [46]. Their study suggested that digital triaging might not contribute to quality improvements and, instead, poses a risk to quality gains. In primary healthcare clinic A, the digital patient management platform was perceived to improve efficiency when accessibility was prioritised over continuity of care (a team of physicians attending patients’ requests instead of their ‛designated’ primary physician). However, this approach may come at a cost to quality gains, as continuity of care is considered to be essential in reducing the risk of patients receiving incorrect diagnoses and treatments [47].

Moreover, it is worth noting a study conducted in 2003 regarding the implementation of a triage-based email system in primary healthcare [48]. The study revealed that email triage did not replace phone communication and despite the increased utilisation of emails, it did not enhance the efficiency of primary care, as the phone volume remained unchanged. Interestingly, even though clinic A reported improved efficiency resulting from the digital triage platform, they continued to experience the same volume of phone calls as before its implementation.

### Strengths and limitations

One strength of this study is its exploration of three different clinics implementing the same patient management digital platform over three years. This multi-case approach enables comparisons and cross-case analysis, which is not very common in the field. The longitudinal nature of the study allowed us to explore the implementation as a process over time rather than at a single point in time. A bottom-up decision-making process involving employees in decisions about work related changes and digitalisation appeared to augment the usage and utilisation of the digital patient management platform. A noteworthy finding was the lasting impact of the first impressions: employees’ initial willingness to adopt and use the platform persisted throughout the study at clinic A, while initial negativity endured and spread to new employees at clinics B and C. Another interesting observation was the substantial influence physicians had on the implementation and utilisation of the platform. At clinic A, one physician’s enthusiasm positively influenced both physicians and nurses at the clinic, whereas hesitation among physicians at clinics B and C seemed to affect the platform’s overall legitimacy. Further research could explore the power balance and the impact that physicians and other professional groups have on the digitalisation of primary care.

This study has several limitations. First, qualitative methods such as interviews and observations depend on interactions between interviewees and the researchers, which can affect the findings. Second, despite inviting all employees from the three clinics, we did not achieve a homogeneous sample representative of the clinics’ workforce, potentially limiting the generalisability to all healthcare professionals working with digital patient management. Third, the study spanned over prepandemic, pandemic and postpandemic periods, with Sweden’s unique pandemic response potentially affecting generalisability [32]. Future research could conduct cross-cultural studies to explore the influence of cultural and contextual factors on the implementation and utilisation of digital patient management. Fourth, focusing on a single digital patient management platform means that user experiences are mediated by this specific platform’s materiality and different platforms might yield different results. Comparative studies of different digital patient management platforms would also be valuable to identify how different platforms influence user experiences and outcomes. However, concentrating on one specific platform, as done in this study, allows for deeper analysis of the findings compared to a more scattered area of study including several platforms. Fifth, the focus on healthcare professionals’ working environment provides a one-sided view, excluding the patient perspective. Future research could incorporate the patient perspective to provide a more holistic understanding of the impact of digital patient management platforms on accessibility and continuity of care. Finally, while our research team comprised researchers from diverse academic fields, including psychology, organisational studies, informatics and human-computer interaction, which strengthened the analysis and flexibility of interpretation, we did not explore quantitative perspectives such as budget and cost efficiency. Future research may benefit from using mixed methods to integrate both qualitative and quantitative methods, providing an even more comprehensive understanding of digital patient management.

## Conclusion

Viewed through the lens of sociotechnical systems theory, achieving harmonious interaction between social and technical components is essential for the successful utilisation of digital patient management. In this context, management can be considered a technical component, given its role in orchestrating and overseeing the structural and operational aspects of the organisation, while healthcare professionals and patients are integral social components within the social fabric of primary healthcare clinics.

Notably, the management and employees at clinic A successfully converged on the platform’s purpose and aligned it with their workflows and logics of care. Clinic A’s approach actively involved employees in the implementation, which enhanced the integration of technical and social elements. Close collaboration with the eHealth service provider allowed for customisation and feedback, ultimately improving alignment with employee needs. The use of interactive, onsite training ensured effective onboarding and utilisation of the digital patient management platform. Clinic A’s proactive patient onboarding and high patient volume demonstrated the crucial role of patient involvement in digital patient management utilisation. However, it is worth noting that an overemphasis on digital patient management may have negative implications for the quality of care in complex cases. Overall, clinic A’s approach empowered the social components, i.e., their employees, resulting in greater utilisation of the platform compared to clinics B and C.

In contrast, clinics B and C lacked coherence. Employees in these clinics questioned the objectives and struggled with the utilisation of the digital patient management platform. The management’s top-down approach disrupted the sociotechnical balance, diminishing employee involvement and hindering platform utilisation. The lack of collaboration with the eHealth service provider seemed to further hinder alignment with clinic-specific needs. The use of mass online training and the absence of proactive patient onboarding limited the platform’s impact on care work. Inconsistencies in working with the platform created confusion and additional work. In summary, the findings indicate that the implementation approaches in Clinics B and C may have disrupted the sociotechnical equilibrium by reducing the role of social components, particularly employees, which consequently restricted the utilisation of the digital patient management platform.

### Electronic supplementary material

Below is the link to the electronic supplementary material.

Supplementary Material 1

## Data Availability

The datasets generated and analysed during the current study are not publicly available due to that the participants in this study did not giving written consent for their data to be shared publicly. However, some of the data are available from the corresponding author (susanne.frennert@design.lth.se) on reasonable request and with permission from the Swedish Ethical Review Authority.
