# Supplementary material for: Unveiling the heterogeneous utilisation of the same digital patient management platform: case studies in primary healthcare in Sweden
Source: BMC Health Serv Res. 2024 Jul 22;24:831. doi: 10.1186/s12913-024-11287-3 (PMC11264364; doi:10.1186/s12913-024-11287-3)
Supplement: Supplementary file 1 — Supplementary Material 1 [file 12913_2024_11287_MOESM1_ESM.docx]

Appendix A

# Interview guides

## Interview Guide Baseline/Round 1 (Before Implementation)

1. What is your role at the clinic?
2. How long have you worked as a 'nurse/doctor/other'?
3. How long have you worked at this workplace?
4. Could you describe your work for me?
5. How many patients do you handle during a workday?
6. Can you describe who is involved in the healthcare process?
7. How interested are you in using digital systems?
8. What advantages do you generally see with digitization in the health care sector? Are there any disadvantages?
9. What advantages do you see with digitization in your area/primary care? Are there any disadvantages?
10. Do you have any thoughts on how XXX PLATFORM will affect the work in the clinic – in what way?
11. Do you have any thoughts on how XXX PLATFORM will affect your work – in what way?
12. Do you have any thoughts on how XXX PLATFORM will affect the patients using the system – in what way?
13. Do you feel that you manage to accomplish what you need to do? Please elaborate and provide examples.
14. Do you feel that there is decision-making space in your work? Please elaborate and provide examples.
15. Do you feel that you have control over what is expected of you in your work? Please elaborate and provide examples.
16. How do you decide how to prioritize among your tasks?
17. How do you perceive social support from your colleagues in your daily work?
18. In what way do you collaborate with other individuals in the clinic?
19. How do you perceive the communication with patients?
20. Do you think that XXX PLATFORM will change the relationships among healthcare professionals in the clinic?
21. The idea behind XXX PLATFORM is for patients to contact the clinic digitally and input health data for triage. Do you think this will affect the patients' relationship with healthcare professionals?
22. What does the digital environment look like at the clinic? – What digital systems do you have? – What are they used for, when are they used, and by whom are they used? – Which of these systems do you work with?
23. Do you feel comfortable starting to work in XXX PLATFORM? (The question is framed differently depending on the person and situation.)
24. Have you received information on how XXX PLATFORM works technically? – Has the information been effective, and if not, how could it have been done differently?
25. Have you seen what the interface looks like for patients?
26. How do you assess your own competence to work digitally?
27. Do you feel confident using digital systems in your work?
28. What are your expectations for XXX PLATFORM?
29. How do you think it will change your work?
30. In what way do you think XXX PLATFORM will change your work environment?
31. Is there anything else that you can think of related to XXX PLATFORM (ONLINE HEALTHCARE)/digitization and your work that you feel we haven't covered?

## Interview Guide Round 2 (Right After Implementation)

### Introduction

1. What is your role at the clinic?
2. How long have you worked as a 'nurse/doctor/other'?
3. How long have you worked at this workplace?
4. Could you describe your work for me?
5. How many patients do you handle during a workday?
6. Can you describe who is involved in the healthcare process?
7. How interested are you in using digital systems?
8. What advantages do you generally see with digitization in the health care sector? – Are there any disadvantages?
9. What advantages do you see with digitization in your area? – Are there any disadvantages?

### About the implementation of XXX PLATFORM

1. Can you describe why you introduced XXX PLATFORM at the clinic?
2. How did you experience the implementation of XXX PLATFORM (positive/negative experiences)?
3. Were you involved or provided input at any stage?
4. Did you receive adequate training/information on XXX PLATFORM before implementation?
5. Did you have any expectations or concerns about the implementation of XXX PLATFORM?
6. What is your experience with the XXX PLATFORM?
7. Can you tell me how XXX PLATFORM works?
8. How has XXX PLATFORM affected the work in the clinic?
9. How has XXX PLATFORM affected your work?
10. How has XXX PLATFORM affected the patients using the system?
11. How do you feel about managing your workload?
12. How do you perceive decision-making space in your work?
13. How do you feel about having control over what is expected of you in your work?
14. How do you decide how to prioritize among your tasks?
15. How do you feel about social support from your colleagues in your daily work?
16. In what way do you collaborate with other individuals in the clinic?
17. How do you feel about communication with patients?
18. Has XXX PLATFORM changed the relationships among healthcare professionals in the clinic?
19. The idea behind XXX PLATFORM is for patients to contact the medical center digitally and input health data for triage. Do you think this will affect the patients' relationship with healthcare professionals?
20. Do you have any suggestions to improve XXX PLATFORM, to make the e-service more user-friendly? – for patients? – for healthcare providers?
21. Do you feel comfortable working in XXX PLATFORM/Do you feel confident with XXX PLATFORM? (The question is framed differently depending on the person)
22. How do you receive information about updates and changes in XXX PLATFORM technical features? – Has the information been effective, and if not, how could it have been done differently?
23. Have you seen what the interface looks like for patients?
24. How do you assess your own competence to work digitally?
25. Do you feel confident using digital systems in your work?
26. How has XXX PLATFORM changed your work environment?
27. Do you encounter any problems when working with XXX PLATFORM? Please elaborate.
28. Who do you turn to for help with problems in XXX PLATFORM?
29. Are there any aspects of XXX PLATFORM that you find unnecessarily complicated?
30. What is the best thing about working with XXX PLATFORM?
31. What is the worst thing about working with XXX PLATFORM?
32. Do you have discussions about XXX PLATFORM at the clinic? – In formal meetings? – Informal meetings?
33. Has working in XXX PLATFORM affected your attitude towards digitization in healthcare? – In what way?

### Conclusion

1. Do you have any recommendations for other organizations about to implement digital healthcare?
2. Is there anything else that you can think of related to XXX PLATFORM and your work that you feel we haven't covered?

## Follow-up Interview Guide Round 3

### Introduction

1. What is your role at the clinic?
2. How long have you worked as a 'nurse/doctor/other'?
3. How long have you worked at this workplace?
4. Could you describe your work for me?
5. How many patients do you handle during a workday?
6. Can you describe who is involved in the healthcare process?
7. How interested are you in using digital systems?
8. What advantages do you generally see with digitization in the health care sector? – Are there any disadvantages?
9. What advantages do you see with digitization in your area? – Are there any disadvantages?

### Implementation of XXX PLATFORM

1. Can you describe why you introduced XXX PLATFORM at the clinic?
2. How did you experience the implementation of XXX PLATFORM (positive/negative experiences)?
3. Were you involved or provided input at any stage?
4. Did you receive adequate training/information on XXX PLATFORM before implementation?
5. Did you have any expectations or concerns about the implementation of XXX PLATFORM?
6. What is your experience with the XXX PLATFORM?
7. Can you describe how XXX PLATFORM has affected the work in the clinic?
8. Can you describe how XXX PLATFORM has affected your work?
9. Can you describe how XXX PLATFORM has affected the patients using the system?
10. What responsibility do you have for your patients?
11. How do you feel about managing your workload?
12. How do you perceive decision-making space in your work?
13. How do you feel about having control over what is expected of you in your work?
14. How do you decide how to prioritize among your tasks?
15. How do you feel about social support from your colleagues in your daily work?
16. In what way do you collaborate with other individuals in the clinic?
17. How do you feel about communication with patients?
18. Has XXX PLATFORM changed the relationships among healthcare professionals in the clinic?
19. The idea behind XXX PLATFORM is for patients to contact the medical center digitally and input health data for triage. Do you think this will affect the patients' relationship with healthcare professionals?
20. Do you have any suggestions to improve XXX PLATFORM, to make the e-service more user-friendly? – for patients? – for healthcare providers? Technology
21. Do you feel comfortable working in XXX PLATFORM/Do you feel confident with XXX PLATFORM? (The question is framed differently depending on whether the person works in XXX PLATFORM or not)
22. How do you receive information about updates and changes in XXX PLATFORM technical features? – Has the information been effective, and if not, how could it have been done differently?
23. Have you seen what the interface looks like for patients?
24. How do you assess your own competence to work digitally?
25. Do you feel confident using digital systems in your work?
26. In what way has XXX PLATFORM changed your work environment?
27. Do you encounter any problems when working with XXX PLATFORM? Please elaborate. – Who do you turn to for help with problems in XXX PLATFORM?
28. Are there any aspects of XXX PLATFORM that you find unnecessarily complicated?
29. What is the best thing about working with XXX PLATFORM?
30. What is the worst thing about working with XXX PLATFORM?
31. Do you have discussions about XXX PLATFORM at the clinic? – In formal meetings? – Informal meetings?
32. Has working in XXX PLATFORM affected your attitude towards digitization in healthcare? – In what way?

### Conclusion

1. Do you have any recommendations for other organizations about to implement digital healthcare?
2. Is there anything else that you can think of related to XXX PLATFORM and your work that you feel we haven't covered?
